# Eyespot variation and field temperature in the Meadow Brown butterfly

**DOI:** 10.1002/ece3.10842

**Published:** 2024-01-17

**Authors:** Sophie Mowbray, Jonathan Bennie, Marcus W. Rhodes, David A. S. Smith, Richard H. ffrench‐Constant

**Affiliations:** ^1^ Center for Ecology and Conservation University of Exeter in Cornwall Penryn UK; ^2^ Center for Geography and Environmental Studies University of Exeter in Cornwall Penryn UK; ^3^ Natural History Museum Eton College Windsor UK

**Keywords:** butterfly, eyespot, phenotypic plasticity, polymorphism

## Abstract

Since the classic work of E.B. Ford, explanations for eyespot variation in the Meadow Brown butterfly have focused on the role of genetic polymorphism. The potential role of thermal plasticity in this classic example of natural selection has therefore been overlooked. Here, we use large daily field collections of butterflies from three sites, over multiple years, to examine whether field temperature is correlated with eyespot variation, using the same presence/absence scoring as Ford. We show that higher developmental temperature in the field leads to the disappearance of the spots visible while the butterfly is at rest, explaining the historical observation that hindwing spotting declines across the season. Strikingly, females developing at 11°C have a median of six spots and those developing at 15°C only have three. In contrast, the large forewing eyespot is always present and scales with forewing length. Furthermore, in contrast to the smaller spots, the size of the large forewing spot is best explained by calendar date (days since 1st March) rather than the temperature at pupation. As this large forewing spot is involved in startling predators and/or sexual selection, its constant presence is therefore likely required for defence, whereas the disappearance of the smaller spots over the season may help with female crypsis. We model annual total spot variation with phenological data from the UK and derive predictions as to how spot patterns will continue to change, predicting that female spotting will decrease year on year as our climate warms.

## Graphical abstract

Eyespot variation in the Meadow Brown butterfly has been held up as a classic example of a genetic polymorphism. Here we show that eyespot variation in the United Kingdom is correlated with field temperatures during larval/pupal development and predict that warmer summers will lead to less spotting. This suggests that thermal plasticity plays a strong role in this system.

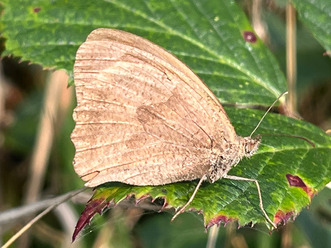

## 1 INTRODUCTION

E.B. Ford defined the term genetic polymorphism as the coexistence of multiple genetic forms in a population (Ford et al., 1940). At that time, Ford was interested in the balance between natural selection and genetic drift in maintaining such polymorphisms and he used natural variation in a range of different butterflies to try and study this question. The most intensively examined butterfly was the Meadow Brown, *Maniola jurtina*. In this iconic species, E.B. Ford, W.H. Dowdeswell and R.A. Fisher used numerous studies of eyespot variation in the field to help establish theories around the nascent field of population genetics (Dowdeswell et al., 1949, 1957, 1960; Dowdeswell & Ford, 1955; Dowdeswell & McWhirter, 1967). Most of these studies, however, assumed that both within (intra‐) and between (inter‐) season eyespot variability was associated with genetic variation (Brakefield & van Noordwijk, 1985; Creed et al., 1964; Dowdeswell, 1981). In other words that the butterfly existed as a series of high and low spot morphs with differences in the spotting of each morph being under genetic control. The Meadow Brown butterfly has therefore long been held up as a classic example of a genetic polymorphism. However, as we now know, phenotypic variation is controlled by a balance between genetic and environmental factors (Pfennig et al., 2010; Schneider & Meyer, 2017). The aim of the current study was therefore to re‐examine this classic butterfly polymorphism in the light of field‐based phenotypic plasticity.

Since the classic studies of Ford, several hypotheses have been proposed to explain the differential apparency of high and low spotted morphs throughout the season, including differential predation or infection (Dowdeswell, 1981), and differential rates of morph development controlled by pleiotropic effects of the wing patterning genes involved (Beldade et al., 2002; Brakefield & French, 1995; Brakefield & Shreeve, 1992; Connahs et al., 2019; Dhungel et al., 2016; French & Brakefield, 1995; Iwasaki et al., 2017; Koch et al., 2003; Monteiro et al., 1997a, 1997b; Otaki, 2020; Reed & Serfas, 2004; Sekimura et al., 2015; Zhang & Reed, 2016). However, importantly, other studies hinted that the environment might be important in controlling eyespot variation. First, measurements of spot heritability (*h*) not only differ between the sexes, but *h* is higher at higher developmental temperatures (Brakefield & van Noordwijk, 1985). Secondly, local environmental changes such as stopping grazing (Dowdeswell et al., 1957; Dowdeswell & Ford, 1955), or prolonged drought (Bengston, 1978; Dowdeswell et al., 1960), seemed to unexpectedly change spot patterns, likely due to the associated changes in microclimate.

Phenotypic plasticity can be defined as the ability of individual genotypes to produce different phenotypes when exposed to different environmental conditions, one of which is temperature (Pfennig et al., 2010; Schneider & Meyer, 2017). In insects, temperature‐related plasticity is well documented in the laboratory with increases in temperature either enhancing (Brakefield et al., 1998), or diminishing (Zhang et al., 2020), spot‐like pattern elements. For butterflies, the critical period for determining the development of wing pattern formation is during late larval development or early pupation (Brakefield et al., 1998). However, most studies of butterfly eyespot variation to date come from temperature‐controlled laboratory experiments (Brakefield et al., 1996, 1998) and not from the field. For example, one recent study of the tropical model the Squinting Bush Brown, *Bicyclus anynana*, shows that the temperature during butterfly development controls the size of butterfly eyespots and that these effects differ for different spot mutants (Mateus & Beldade, 2022). Importantly, it was also been noted that such laboratory experiments alone cannot determine the likely effects of field temperatures on the multiple genotypes present in any given population (Rodriguez & Beldale, 2020).

Although the number of eyespots in *M. jurtina* often declines across the season, in so‐called intra‐seasonal variation (Dowdeswell, 1981), the likely role of temperature was not considered. This decline in hindwing spotting in females was therefore again attributed to differential survival of high‐ and low‐spot morphs throughout the season (Brakefield & van Noordwijk, 1985). To examine the role of phenotypic plasticity in this classic polymorphism, here we looked for correlations between the temperature during butterfly development in the field and the number of spots present in the resulting adult. Specifically, we examine the hypothesis that increased temperatures during late larval/early pupal development are correlated with a reduction in the number of spots present on the wings of the resultant adult, thus explaining the decline in spotting across the season as temperatures warm through the summer. This thermal dependence is likely because accelerated rates of scale development do not allow the full development of the eyespot at higher field temperatures, as discussed extensively elsewhere (ffrench‐Constant & Koch, 2003; Koch et al., 2000).

To try and explain the decline in spotting across the season we therefore wanted to specifically repeat the presence–absence spot scoring of Ford and others. However, we also wanted to include larger continuous collections of adults (both males and females) from multiple sites over multiple years, scored by a single observer in the laboratory. Using a single standardised technique overcomes some of the errors in spot scoring previously documented by Brakefield and others when different observers use different spot scoring techniques (Brakefield & Dowdeswell, 1985). Here, we show that the temperature 35 days prior to adult capture best predicts the number of spots found on the adult female butterfly. This developmental model better describes the decline in total spot counts across the season, rather than simply the number of days since 1st of March, because temperature fluctuates within individual summers and does not increase in a linear fashion throughout the season. Finally, we use this model to predict how total spot number will decline at individual sites as our summers warm with climate change. Taken together, our data suggest that eyespot variation in the Meadow Brown is correlated with the temperature in the field at the time of the butterflies' development, this stands in contrast to previous work on this iconic polymorphism which has focused on differential survival of genetically determined high‐ and low‐spot morphs.

## 2 METHODS

### 2.1 Collection and scoring

Butterflies, all of the same sub‐species of *M. jurtina insularis* (Thomson, 1969), were collected from three different sites across the United Kingdom by the authors. First, near the town of Eton in Berkshire (grid reference SU965775 with butterflies collected every year from 1988 to 1993, *n* = 2158 butterflies), collected by David Smith. Second, near Buckingham in Buckinghamshire (grid reference SP687339 from 1988 to 1991, *n* = 788 butterflies), all collected by David Smith. Third, at Chycoose, near Truro, in Cornwall (grid reference SW804390 and year collected 2020, *n* = 1796 butterflies), collected by Richard ffrench‐Constant. Butterflies were collected daily during the warmest part of the day and all specimens were collected regardless of the sex encountered. Butterflies from all three field sites, for all years, were placed in individual envelopes in the field and then ‘side‐set’ as large and continuous series representing daily collections at all sites. Side‐setting means we could readily score all 10 spot positions on each individual while reducing the very large space necessary to store all specimens in a museum for further reference.

**FIGURE 1:**
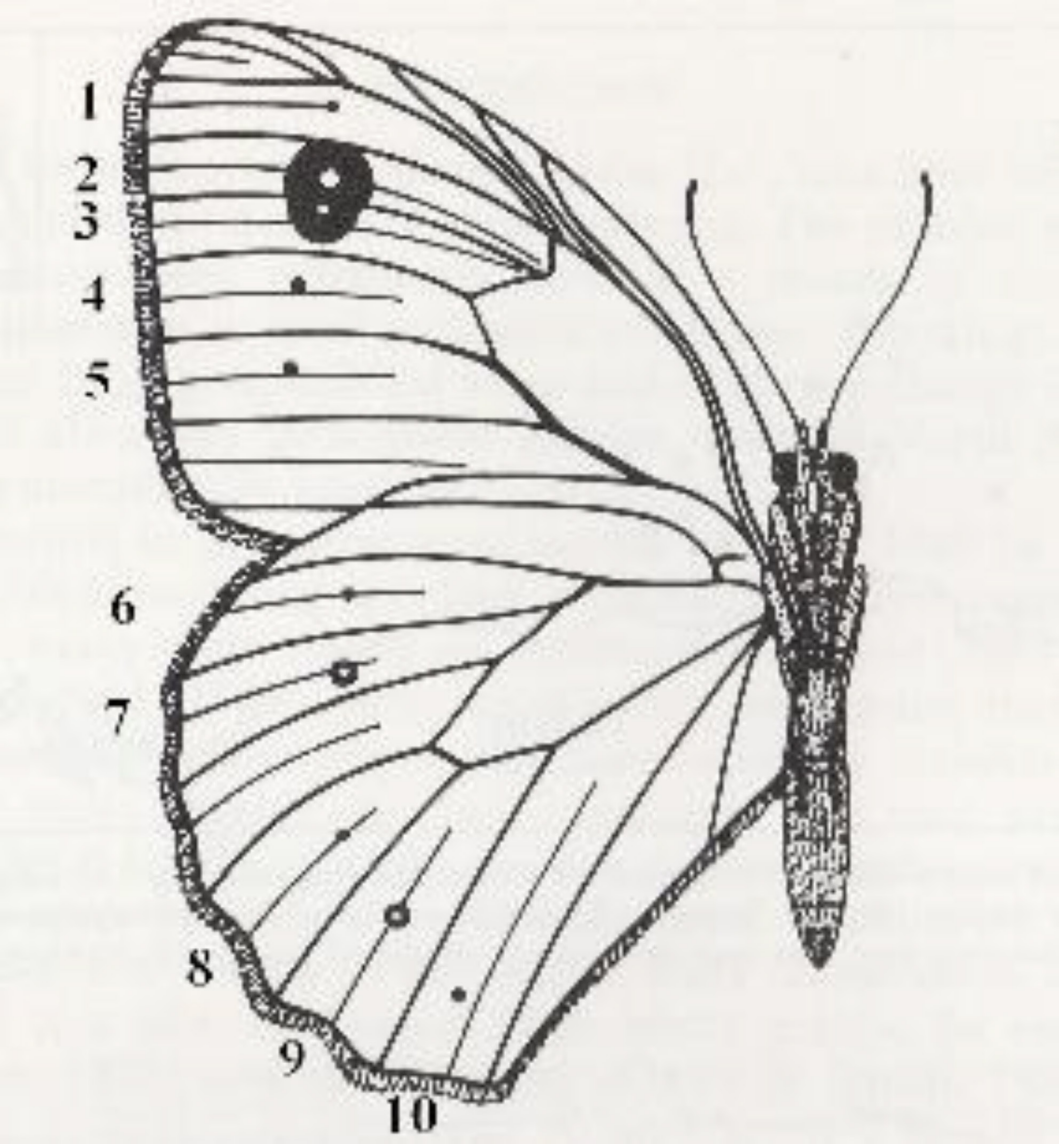
Spot scoring system for the Meadow Brown. Spots 1–5 are on the forewing and spots 6–10 are on the hindwing. Note that spots 2 and 3 are usually fused and are here referred to as compound spot 2/3.

We adopted the following improvements to Ford's original scoring protocol to avoid previous confusion around spot numbering. First, we numbered all spot positions from 1 to 10 (Figure 1). Spot positions were numbered 1–5 on the forewing and positions 6–10 on the hindwing. Spots 2 and 3 on the underside of the forewing are usually joined and were therefore scored as a compound spot, here termed 2/3 (see below). We therefore looked at *all* spots on both the fore‐ and hindwing, in both sexes, and not just the hindwing spots of females scored by Ford and others (Dowdeswell, 1981; Dowdeswell et al., 1949, 1957, 1960; Dowdeswell & Ford, 1952, 1955; Dowdeswell & McWhirter, 1967). Second, all scoring was performed by a single observer (D.A.S), on a single (right hand) side of the butterfly, defining spot presence as the presence of at least one melanic raised scale visible under a 10× hand lens, at the predicted spot location, and the lack of any such raised scale as absence. This ensures a *precise and repeatable* method of scoring spot presence/absence, not previously possible in the field with multiple observers scoring living animals in a butterfly net. Using a single observer (D.A.S.) to derive, the entire data set in the current study therefore removes any possibility of variation previously shown to be introduced by different scorers (Brakefield & Dowdeswell, 1985). Third, we scored a total of 4742 butterflies representing 47,420 spot positions scored at each of the 10 candidate spot positions (spots 1–10, see Figure 1 for spot numbering), this sample size far exceeds any previous studies which have looked at tens or hundreds of butterflies only. Finally, because monthly temperatures have recently been correlated with wing length (or ‘body size’) in several species of UK butterflies (Davies, 2019), which might therefore affect the size of the omni‐present compound spot 2/3, we also measured the forewing length (in mm using Vernier callipers) of the large and continuous series of Cornish females collected from Chycoose Farm, Truro, in the summer of 2020. We then measured both the width and height of the compound forewing spot 2/3 to calculate its size. Width (*a*) and height (*b*) were then used to estimate the total area (*A*) of spot 2/3 using the formula *A = π a b*. All scoring and wing measurements were performed by a single observer, D.A.S.

### 2.2 Field temperature

Daily temperature data were obtained from the UK Meteorological office (MET) for each of the locations and years when specimens were collected, during the flight season between 1st March and 31st October. For Buckingham and Eton, HadUK‐Grid data from the 5 km grid square containing each study site was used (Hollis et al., 2019). This data gives the maximum and minimum air temperatures, measured over 24 h; the average of these two daily temperatures was used in the analysis. For the Chycoose Farm, Truro, Cornwall location the 2020 gridded temperature data was not yet available (at the time of analysis), so instead temperature data were used from the closest weather station, located approximately 18 km away, in Camborne.

### 2.3 Variation between sexes

Data analysis was conducted using R version 4.1.2 (R Core Team, 2022) using packages lme4 v.1.1.32 and lmtest 0.9.40. To look at any potential difference between the sexes, a principal component analysis (PCA) of all the data was conducted with the sexes combined. The first principal component was associated with differences between the sexes, confirming what Ford and others observed that variation is indeed greater in females (Dowdeswell & Ford, 1952). The PCA was then repeated using only the females in order to analyse within female variation only. In this female‐specific analysis, the first principal component was correlated with the wing spot total.

### 2.4 The developmental model

**FIGURE 2:**
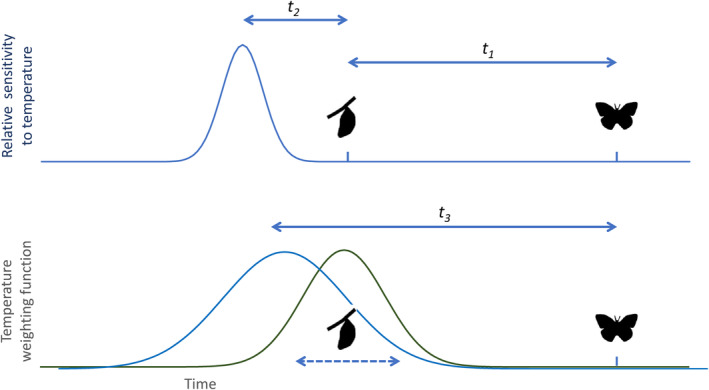
Schematic representation of method used to sample developmental temperature. Top panel: a sampled butterfly on a particular day has an unknown time *t*_1_ since its pupation. The period of peak sensitivity to temperature is an unknown time *t*_2_ prior to pupation. We assume that relative temperature sensitivity curve follows a Gaussian curve (with unknown standard deviation) over time. Bottom panel: in our observations, the date of pupation is unknown (dashed arrow), but we assume that the age of adult butterflies on any particular date also follows a Gaussian distribution. The probability density function of the true pupation date (green) thus also follows a Gaussian curve as does our temperature weighting function (blue), which is the sum of the temperature weighting and the probability density function of pupation date. The mean time lag between adult observation and most probable peak sensitivity (*t*_3_), and the standard deviation of the weighting curve, are both fitted to observations.

A general linear mixed model (or GLMM), with a Gaussian error distribution, was used to test whether the specimens showed intra‐seasonal shifts in wing spot patterns. The model included the wing spot total as the response variable, and the calendar (Julian) date of specimen collection and location as explanatory variables, and year as a random factor. The significance of each of these explanatory variables was tested using likelihood ratio tests. In other species, it has been shown that wing phenotypes are determined during the late instar larvae and early pupation (Beldade & Monteiro, 2021). The specimens used in this study were all caught in the field so the exact timing of their pupation cannot be determined. However, in the Meadow Brown pupation is not synchronous and larvae continue to pupate throughout the two‐to‐three‐month flight season. Therefore, it was necessary to identify the number of days prior to date of capture (the lag time) that the temperature best explains wing spot patterns (see Figure 2 and caption, for a description of the assumptions underlying our approach). Different estimated developmental temperatures were used in modelling, with lag times ranging from 1 to 75 days, sampled according to a Gaussian weighting with standard deviations of between 1 and 10 days. GLMs with wing spot total as the response variable, and developmental temperature, days since 1st March and location as explanatory variables were run. The Akaike Information Criterion (AIC) values of these different models were compared, and the lag time and standard deviation from the model with the lowest AIC value was used to determine the estimated developmental temperature for all subsequent analyses.

### 2.5 Developmental temperature and spot pattern

To confirm whether estimated developmental temperature is a more parsimonious predictor of spot pattern than day of the year (and hence whether we can be confident that temperature, rather than other factors that vary throughout the season are likely drivers of spot pattern), we ran linear mixed effect models with the first principal component of female spot pattern as the response variable and combinations of explanatory variables including estimated developmental temperature (derived from the process described above), day of the year and location. Year was included in the analyses as a random effect in this set of models. Model simplification, using likelihood ratio tests, was conducted to identify the minimum adequate model explaining variation in the first principal component of wing pattern. Following these analyses, a predictive model of total spot number, derived from a GLM with wing spot total as the response variable and the coefficients of the most parsimonious set of explanatory variables found in the previous analysis was derived.

Generalised linear mixed effect models were also used to test the effect of developmental temperature on the presence or absence of each of the individual wing spots. Each wing spot was modelled separately, with wing spot presence or absence as the response variable, developmental temperature as a fixed effect, and year as a random effect. A binomial error structure was used, as wing spot presence or absence is a binary variable. Location and days since 1st March were not included as fixed effects, as the results of the prior analysis on overall wing spot pattern found them to be non‐significant. Sigmoidal plots were then produced for each of the 10 wing spots, plotting the fixed effects of the model.

### 2.6 Predicted spatial and temporal trends in spot patterns

In order to examine the likely effects of climate and phenology across the United Kingdom on spatial and temporal variation in spot pattern, we looked at transect data obtained from the Butterfly Monitoring Scheme (BMS). The BMS has butterfly abundance data from over 2500 transects collected since 1976. In these transects, all butterflies within 2.5 m either side of the transect line and 5 m ahead of the surveyor are recorded. The data used from the BMS included the location of each transect, the date it was walked, and the total count for the number of *M. jurtina* observed. Due to inconsistencies in the longevity of different transects, and the frequency of repeated sampling, the full data set was filtered and 20 transects (Table 1) were selected. The selected transects were all established prior to 1981, and were walked regularly each year, allowing significant temporal patterns to be identified across the United Kingdom.

**TABLE 1:** 20 selected transects from the BMS data and their locations.

Site number	Site name	OS grid reference
6	Yarner Wood	SX770780
10	Oxwich	SS500870
19	Studland Heath	SZ020790
23	Ebbor Gorge	ST528488
28	Walberswick	TM470740
29	Aston Rowant (N)	SU730960
34	Potton Wood	TL250500
45	Gibralter Point	TF560580
47	Skomer	SM729093
54	Nagshead	SO600090
116	Taynish	NR720820
60	Lindisfarne	NU130430
61	Insh Marshes	NH810010
62	Northward Hill	TQ780760
72	Gait Barrows	SD470770
84	Ampfield Wood	SU410238
85	Derbyshire Dales	SK180650
95	Wyre Forest	SO755766
97	Lullington Heath	TQ540020
100	Ynys Hir	SN670950

The BMS count and date data were used to test whether there is any evidence of phenological differences in *M. jurtina* flight periods across the United Kingdom. If *M. jurtina* are adjusting their phenology in different parts of the country, this will affect the developmental temperatures that butterflies are exposed to, and therefore will affect the expected spatial patterns of wing spot totals. The selected transects were divided into ‘North’, ‘Southwest’ and ‘Southeast’ based on their location.

To model the likely number of eyespots, the predicted developmental temperatures for each of the BMS transect records was calculated using the previously identified lag time and standard deviation from the sliding window analysis. For each of the 20 transects, 5 km resolution HadUK‐Grid temperature data were used. Using this estimated developmental temperature, the wing spot total for each butterfly observed was predicted using the wing spot total model created using the Buckingham, Eton and Truro data. The predicted wing spot totals of the butterflies were then calculated from the developmental model and plotted against the days since 1st March, for the ‘North’, ‘Southwest’ and ‘Southeast’ localities separately. Linear models were subsequently used to statistically test the significance of any change in predicted wing spot totals intra‐seasonally.

To examine how eyespot variation changes over time and space the annual average predicted wing spot total for each of the transects was calculated; this mean was weighted by the number of *M. jurtina* counted on each of the individual transect walks within that year. To examine inter‐seasonal patterns in predicted wing spot totals, the Lullington Heath transect (Ordnance Survey grid reference SQ540020) was used, on the basis that it was walked more frequently than any of the other selected transects (*n* = 668, from 1979 to 2019). The mean annual average developmental temperatures were calculated; this mean was weighted by the count of *M. jurtina* to reflect the developmental temperatures experienced by most of the population. Data for the average summer temperature were obtained from the MetOffice, to provide an indication of the general conditions each year. This seasonal summer temperature is the mean temperature value between June and August, extracted at a 5 km resolution for each of the years the transect was walked. Linear mixed models were used to test the relationship between the annual average predicted wing spot total and the seasonal summer temperature; the seasonal summer temperature and annual average developmental temperature; the seasonal summer temperature and the year; the annual average predicted wing spot total and the year or if they are exclusive. Transect ID was included in each of these models as a random factor. The analysis of the effect of the seasonal summer temperature on annual average predicted wing spot total, and the effect of the seasonal summer temperature on annual average developmental temperatures, was also repeated for the dataset with all the 20 transects, to test whether the observed patterns are a generality, or if they exclusive to the Lullington Heath transect. In this analysis, the seasonal summer temperatures will also reflect geographic differences, because multiple transects are used. This seasonal summer temperature was extracted for each transect location at a 5 km resolution, for each of the years the transect was walked. For the transect at Lindisfarne, 5 km was not a suitable resolution, so temperature data at a 1 km resolution was used instead.

The size of individual spots, and hence the detection of individual spots by observers, might be considered to be a function of the wing length of the butterfly. To test a possible relationship between wing length, spot size and developmental temperatures, the wing length and size of the compound spot 2–3 were measured in the individuals collected at Chycoose, Cornwall. Relationships between estimated developmental temperature, day of the year, wing length and spot size were investigated using linear models between pairs of variables.

## 3 RESULTS

### 3.1 The developmental model and total spot count

**FIGURE 3:**
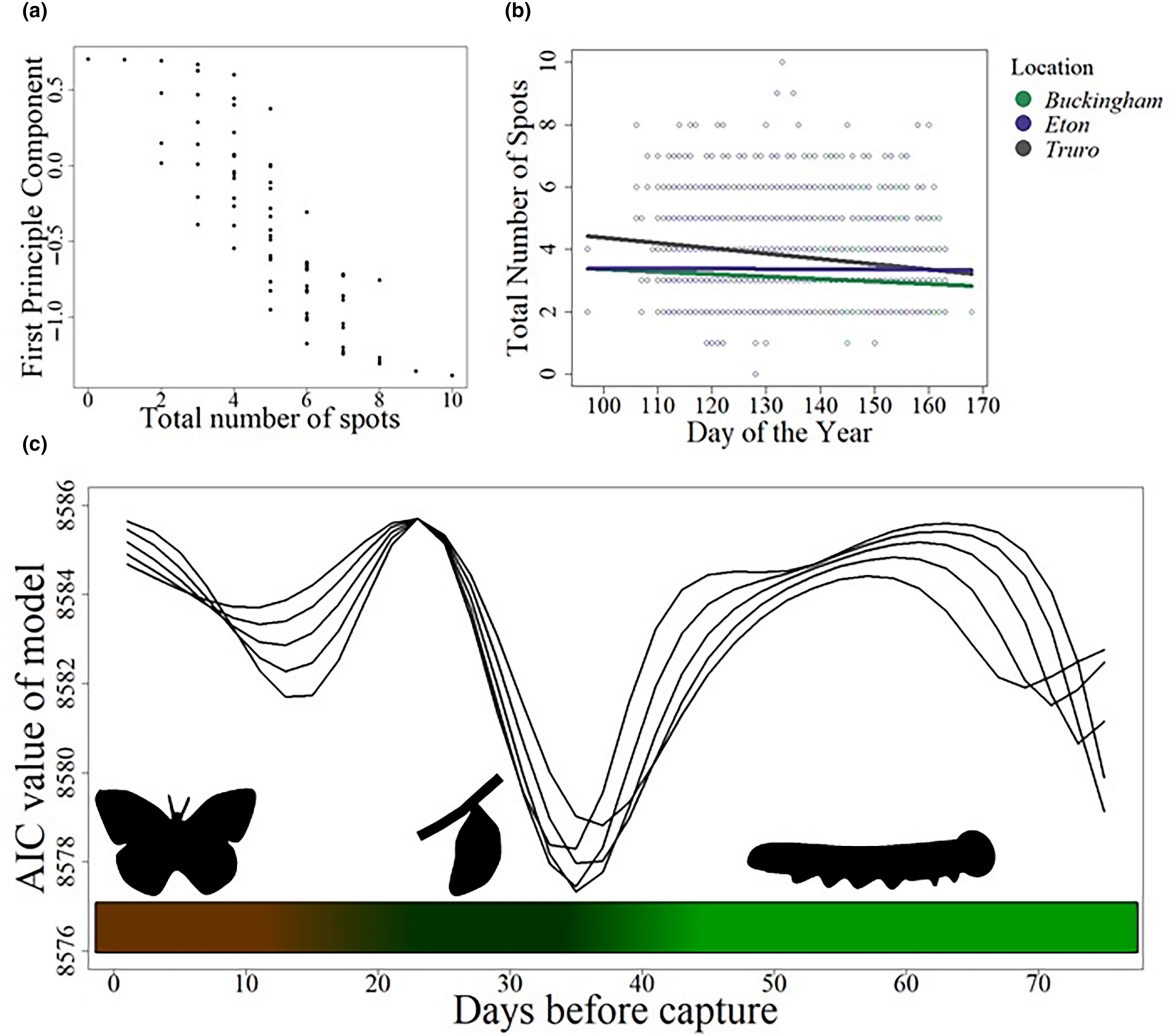
Developmental temperature correlates with the decline in female spottiness within a season. (a) Principal components analysis shows that the first principal component is correlated with total number of spots. (b) The total number of female spots declines through the season at all three sites (Eton, Buckingham and Truro). Day of the year is the number of days since the start of the flight season on 1st March. (c) The Akaike information criterion (AIC) results from the models run lag times (days before specimen capture) of between 1 and 75 days, and standard deviations between 6 and 10 (shown as separate lines). The model was run with total number of spots as the response variable, and location, day of the year and developmental temperature, at the given lag and standard deviation, as the explanatory variables. The model with the lowest AIC value had a lag of 35 days with a standard deviation of 8. The coloured bar at the bottom of the plot reflects the likely life stages of the butterfly at each of the lag times, with brown showing the adult stage, dark green showing the pupal stage (~28 days) and light green showing the larval stage. Data for female butterflies only, from all sites combined (*n* = 2504 butterflies).

**FIGURE 4:**
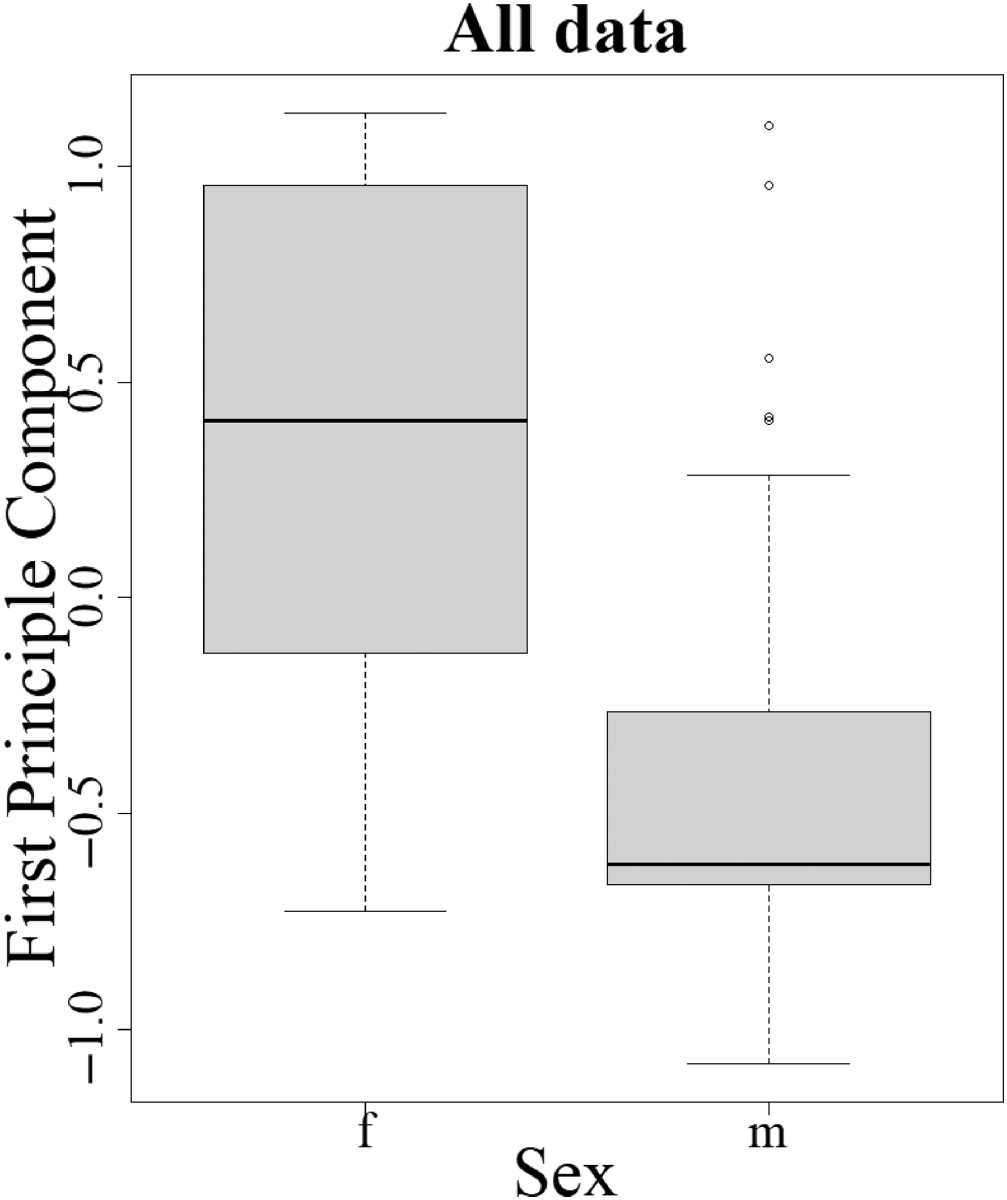
Principal component analysis of all spot data shows significant differences between males and females. This is consistent with the hypothesis that female eyespots are more plastic than those of males and confirms why Ford and others only scored variation in females (see text for discussion).

**FIGURE 5:**
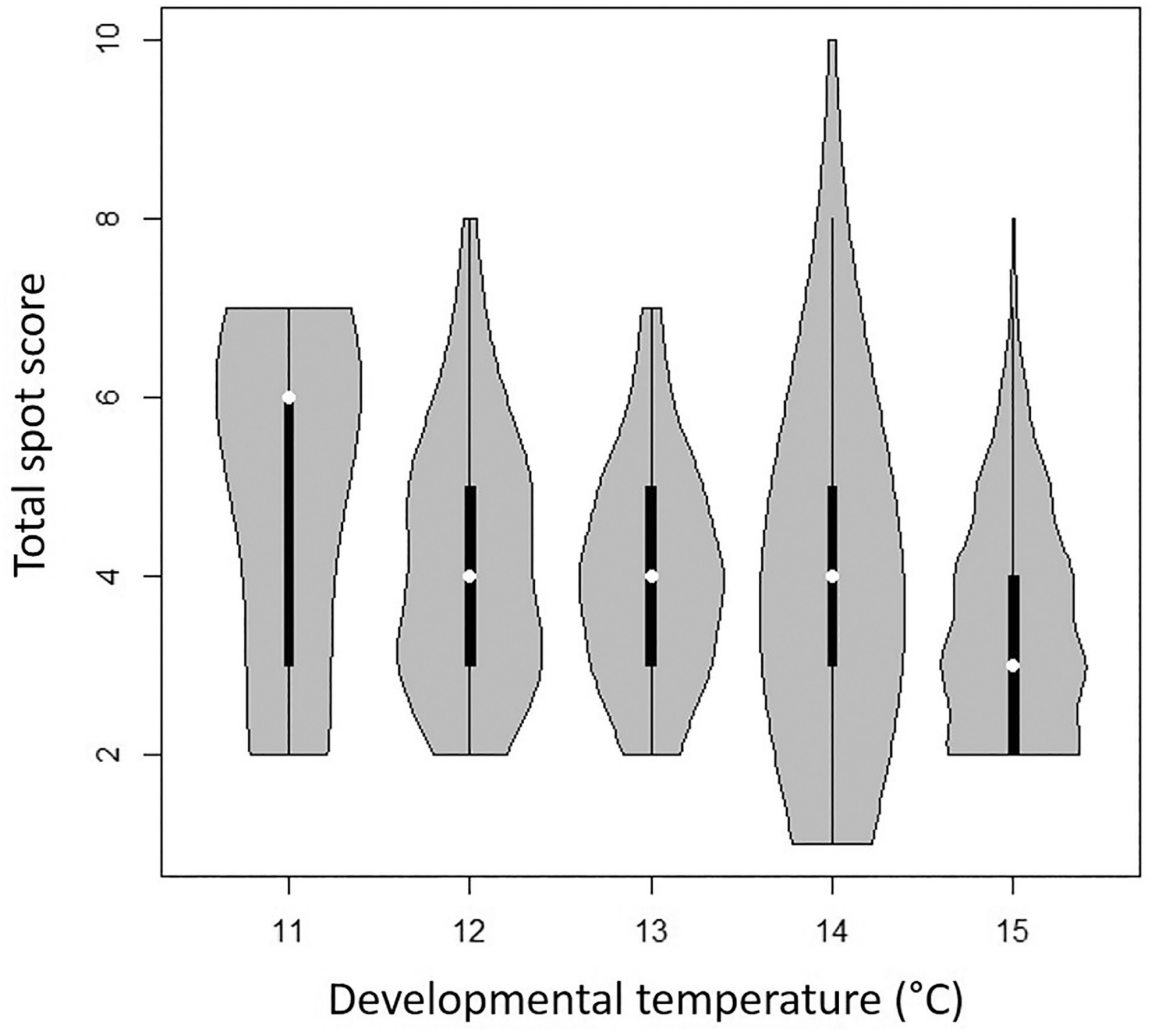
Violin plot of total spot score for all Cornish females collected from Chycoose Farm in 2020, displayed by developmental temperature (rounded to nearest °C). Temperature data have been rounded to the nearest integer and grouped together for clarity. Note that higher developmental temperature leads to a lower spot score in the adult butterfly, with females developing at 11°C having a median of six spots and females at 15°C having only three.

To examine the effects of temperature on female eyespot variation we first confirmed the original observations of Dowdeswell and Ford, that spot numbers (*n* = 2504 individual butterflies) do indeed decline across the season at all three sites (GLMM: $\chi_{7,2}^{2}$ = 28.88, *p <* .001, Figure 3) in so‐called intra‐seasonal variation (Dowdeswell, 1981). Second, we performed a principal component analysis (PCA) of spot pattern in both sexes combined (Figure 4) which showed significant variation in spot pattern between males and females. We therefore ran a new PCA on females only in order to examine female variation alone. Principal component one (accounting for 39% of the variation) in the female‐only analysis correlates strongly with the total number of spots (Figure 3a; *R* ^2^ = 0.87, *p* < .001). We then used a moving window analysis to find the period during which field temperature best‐explained spot number variation (see Figure 2 and caption for details of the assumptions underlying this model), weighting temperature using a Gaussian weighting. This analysis shows the most parsimonious model was a sampling weight with a lag of 35 days, weighted by a Gaussian curve with a standard deviation of 8 days (see lowest AIC value in Figure 3c). This model suggests that eyespot variation is related to the temperature around 35 days prior to the capture of the butterfly, at a time consistent with the animal being a late larva or during early pupation (assuming a total of 28 days spent in the pupa (Dowdeswell, 1981; Eeles, 2019) and an estimated mean of ~7 days spent as an adult prior to capture in this study). Here we term this the estimated developmental temperature. To confirm that the effect was directly related to temperature, and not simply correlated to developmental temperature as a proxy for geographical location and/or seasonal changes driven by other factors, we used this estimated developmental temperature in a multi‐model comparison in which developmental temperature was replaced with day of the year as a linear fixed factor, both developmental temperature and day of year were included and/or location was included in the model as a fixed factor, with year as a random factor in every model. In each case the most parsimonious model (lowest AIC value) was that included developmental temperature alone, and when both developmental temperature and day of year were included the developmental temperature term was significant within the model (*p* = .049) while the day of the year was not (*p* = .201). Table 2 shows the relative AIC values of the model set. When developmental temperature alone was included, the effect of this term was highly significant (*p* < .001). To look at correlations of developmental temperature with total spot count, across a single season, we also analysed the large and continuous series of Cornish females collected across the entire 2020 flight season. Violin plots of total female spot score against developmental temperature for this site (Figure 5) show that higher developmental temperature leads to a lower overall spot score, with females developing at 11°C having a median of six spots and those developing at 15°C having only three. This confirms and Ford and Dowdeswell's original observation that spotting in females declines over the season (Dowdeswell, 1981) and strongly suggests that developmental temperature is a proximal causal factor.

**TABLE 2:** Comparison of AICc values for combinations of fixed effects explanatory variables in linear mixed models predicting the first principal component of female spot pattern in *Maniola jurtina.*

Explanatory variables	df	AICc	ΔAIC
Temperature	4	4566.294	0
Temperature + location	6	4570.471	4.177
Day of year	4	4572.316	6.022
Day of year + location	6	4575.353	9.059
Temperature + day of year	5	4576.044	9.75
Temperature + day of year + location	7	4580.486	14.192
Null model (intercept only)	3	4594.492	28.198

*Note*: All models contained year as a random effect. The most parsimonious model contains only temperature (the estimated developmental temperature) as a fixed effect. Day of year was measured as a continuous variable from 1st March; location as a categorical variable (Eton, Buckingham or Cornwall).

### 3.2 Developmental temperature and the presence/absence of individual spots

**FIGURE 6:**
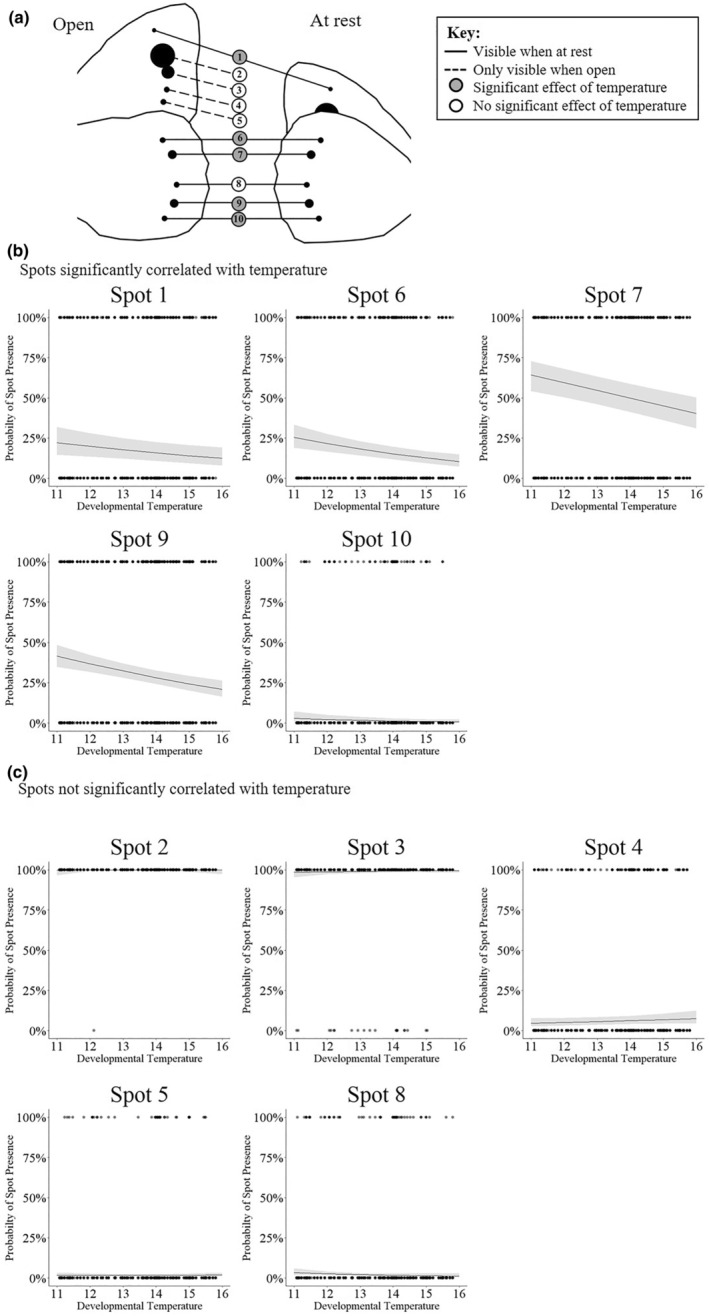
Field temperature determines the probability that wing spots visible in females are present or absent. (a) Visibility of the 10 wing spots (spots 0–10) when the butterfly's wings are open and when at rest. Spot numbers 2, 3, 4 and 5 are not visible when the butterfly is at rest. (b) Presence or absence of spot numbers 1, 6, 7, 9 and 10 are significantly affected by temperature (see text). In all five of the spots, the probability of having the individual spot declines as developmental temperature increases. (c) Presence or absence of spots number 2, 3, 4, 5 and 8 are not significantly affected by temperature. The points show the observed values from the combined Buckingham, Eton and Truro data (*n* = 2504 for each spot scored), the line shows the fixed effects of the model that spot likelihood is affected by developmental temperature, and the grey shading shows the 95% confidence intervals of the model's predictions.

Using this developmental time window (temperature 35 days prior to capture), there is a highly significant effect of developmental temperature on PCA component 1, with higher temperatures leading to fewer spots (*p =* .001). Analysing spot number totals using the same method, there is also a highly significant effect of developmental temperature on both hind‐ and forewing, and total number of spots (*p <* .001; for total number of spots coefficient = −0.099 SE = 0.023). Fascinatingly, if the relationship between individual spots and developmental temperature is plotted, as shown in Figure 6, then variation in five of the six spots that are visible in female butterflies at rest (Figure 6a), namely spots 1 (GLM: $\chi_{3,1}^{2}$ = 7.32, *p =* .006), 6 (GLM: $\chi_{3,1}^{2}$ = 17.6, *p <* .001), 7 (GLM: $\chi_{3,1}^{2}$ = 19.9, *p <* .001), 9 (GLM: $\chi_{3,1}^{2}$ = 21.6, *p <* .001) and 10 (GLM: $\chi_{3,1}^{2}$ = 5.18, *p =* .023) is correlated with temperature (Figure 6b). With increasing developmental temperature leading to a decrease in the probability that each of these visible spots will be present. Whereas, in contrast, all four of the spots hidden at rest (spots 2 (GLM: $\chi_{3,1}^{2}$ = 1.00, *p =* .317), 3 (GLM: $\chi_{3,1}^{2}$ = 1.42, *p =* .233), 4 (GLM: $\chi_{3,1}^{2}$ = 1.81, *p =* .178) and 5 (GLM: $\chi_{3,1}^{2}$ = 0.0038, *p =* .951)) show no significant variation with developmental temperature and neither did spot 8 (GLM: $\chi_{3,1}^{2}$ = 3.18, *p =* .07) (Figure 6c). This temperature‐driven plasticity in female eyespots contrasts sharply with variation in males where only a single eyespot (spot 3) differs significantly with developmental temperature (see Table 3), and most of the remaining variation correlates with sample location and not temperature.

**TABLE 3:** In contrast to females (see also Figure 6), only spot 3 varies with developmental temperature in males and variation in all other male spots is driven by geography.

Spot	*p* Value		
	Developmental temperature	Location	Day of the year
1	.763	<.001*	.735
2	Always present in our samples		
3	.007*	.003*	<.001*
4	.973	.023*	.627
5	.813	.153	.338
6	.413	<.001*	.010*
7	.372	<.001*	.031*
8	.135	.030*	.102
9	.118	.045*	.018*
10	.965	<.001*	.009*

*Note*: The *p‐*value results from the model simplification using likelihood ratio tests are shown. A separate model was run for each spot which included year as a random effect and has a binomial error structure, as spot presence or absence is a binary variable. Spot 2 could not be tested as it is always present as the dominant component of compound spot 2/3. The significant results (*p* < .05) are marked (*). Data are from males combined across all three sites sampled *n* = 2238 butterflies, corresponding to 22,380 spot positions scored.

### 3.3 Wing length and the size of compound spot 2/3

**FIGURE 7:**
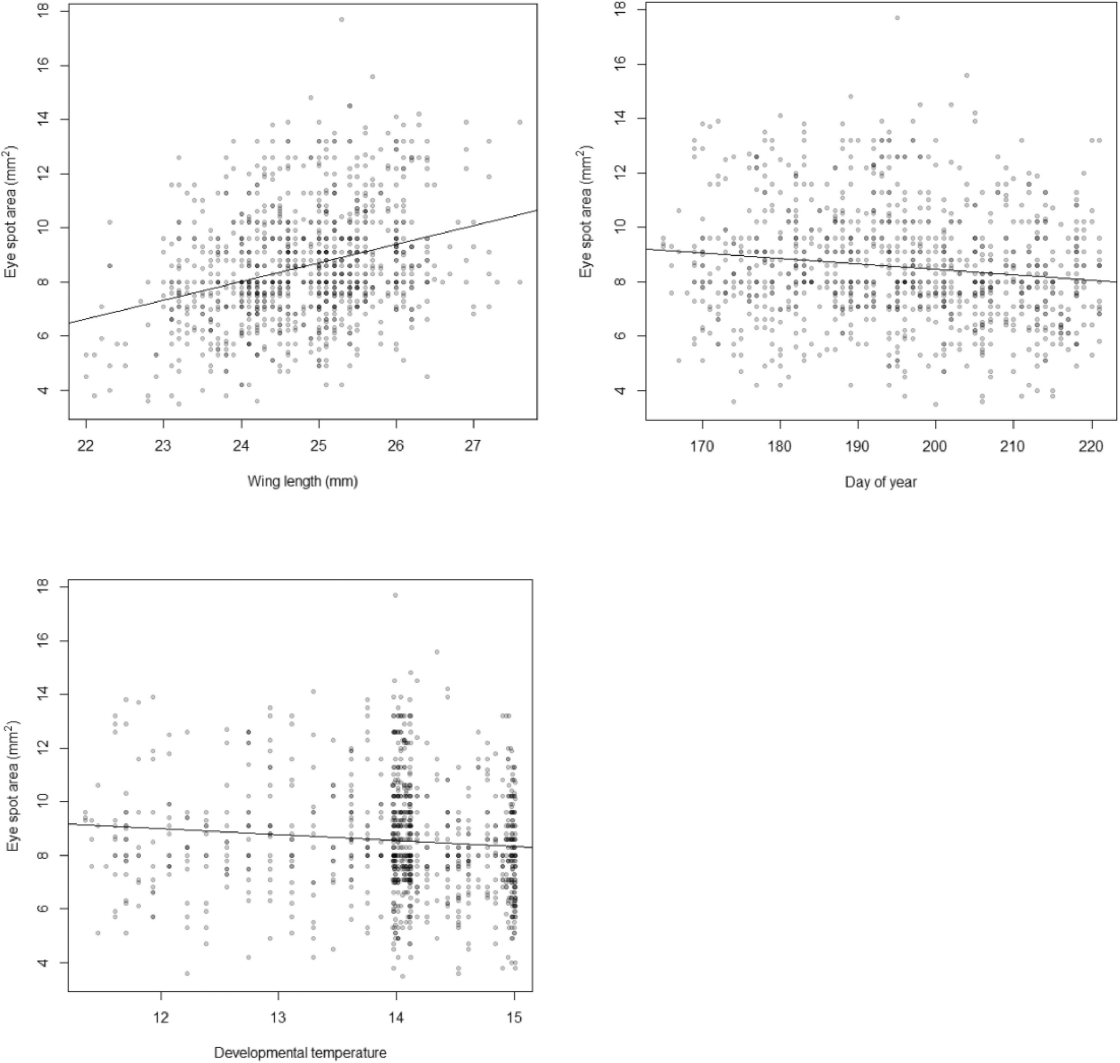
Correlations of the area of eyespot 2/3 with wing length (mm), day of the year and developmental temperature. Note that wing length is positively correlated with the area of eyespot 2/3, while both day of the year and developmental temperature show negative correlations with the total area of this spot (see text for discussion).

To examine effects on spot size, we also examined the effect of wing length on the height, width and estimated total area of the compound (merged) forewing spot 2/3 (Figure 7). This compound spot is usually formed by the merger of spots at wing positions 2 and 3; however, it is still possible to measure the size of spot 3 even when it is merged with the larger spot 2 (see below). We found that forewing length does indeed decrease across the flight season in the large cohort of female butterflies collected from Chycoose, Cornwall in 2020. However, critically, wing length variation is best explained by the days since 1st March (day of the year) rather than developmental temperature which accounts for the variation in the presence/absence of the remaining eight smaller spots. Thus, a linear model predicting wing length as a function of both developmental temperature and day of the year shows a significant effect of day of the year (wing length decreases through the season, *p* < .001) but no effect of developmental temperature (*p* = .254). The partial *r* ^*2*^ (explanatory power) of days since 1st March (*r* ^*2*^ = .001, Table 4 below) is therefore considerably higher than that of developmental temperature (*r* ^*2*^ = .019, Table 4). In turn, the total size (area) of the compound spot 2/3 correlates positively with wing length but negatively with day of the year (Figure 7), suggesting that control of the size of spot 2/3 is independent from the remainder of the smaller spots. Critically therefore, taken together, this data suggests that wing length is controlled independently of spot presence/absence. This further analysis confirms that total spot count (the central character scored by E.B. Ford and others) is correlated with developmental temperature whereas wing length and the corresponding size of the large omni‐present compound spot 2/3 is correlated with day of the year and is therefore controlled independently.

**TABLE 4:** Model statistics for GLM predicting the size of compound spot 2/3 by developmental temperature and day of year

	Coefficient	SE	*t*‐value	*p‐*value	Partial *r* ^*2*^
Intercept	27.892782	0.459468	60.707	<2e−16***	
Developmental temperature	0.094210	0.082596	1.141	.254	.001
Day of year	−0.022635	0.005136	−4.407	1.16e−05***	.019

*p*‐value *** = <0.0001.

### 3.4 The phenology of spot variation across the United Kingdom

**FIGURE 8:**
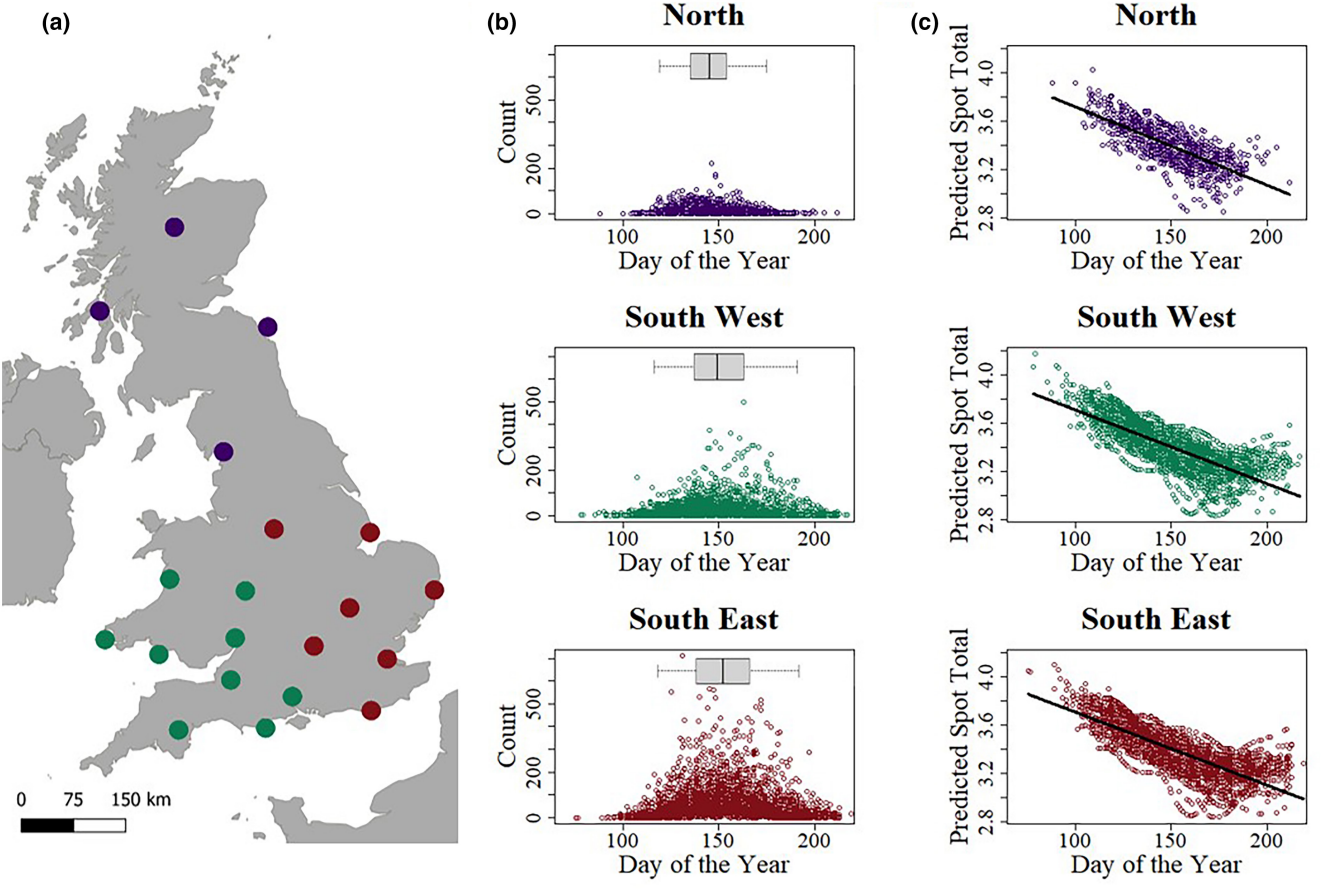
Phenology of the Meadow Brown flight period across the United Kingdom and the corresponding decline in female spottiness predicted intra‐seasonally. (a) Location of the twenty selected BMS transects, with the colours representing whether they were classified as ‘North’ (purple), ‘South West’ (green) or ‘South East’ (red). (b) Number of Meadow Browns observed in each transect walk, against the day of the year the transect was walked, for each of the localities. The day of the year is the number of days since the start of the flight season on 1 March. The box plots above each plot (in panel b) show the quantiles of the day of the year data. The central line is the 50% quantile, the edges of the box are the 25 and 75% quantile, and the whiskers are the 2.5 and 97.5% quantiles. (c) Predicted spot totals change with day of the year at each of the localities. The predicted spot total declines intra‐seasonally in all three localities.

Given that spottiness in females declines with increasing developmental temperature in the field, we wanted to examine if the Meadow Brown can effectively compensate for such changes in temperature by altering its flight phenology across its range. The phenology of the flight season differed between the localities sampled but only towards the end of the season (Figure 8b). The timing of peak population sizes was therefore relatively consistent between sites (the 50% quantile varies from day 145 to 152 between the localities), as was the start of the flight season (2.5% quantile varies from Day 116 to 119 between the localities), consistent with previous studies (Brakefield, 1987). But the timing of the end of the flight season, however, varies significantly between locations, being earlier in the Northern transects (97.5% quantile is day 175 for the North transects, compared to Day 191 and day 192 for the Southeast and Southwest, respectively). This means that the length of the flight season is shorter in the more northerly transects; the 95% range for the North transects is 56 days, compared to 74 days and 75 days for the Southwest and Southeast respectively. Finally, we used this data to predict female spot number variation in the 20 sampled transects. The predicted wing spot total declined significantly with day of the year in the North (GLM: $\chi_{3,1}^{2}$ = 665.8, *p <* .001; Figure 8c), Southwest (GLM: $\chi_{3,1}^{2}$ = 2701.6, *p <* .001; Figure 8c) and Southeast (GLM: $\chi_{3,1}^{2}$ = 2582.8, *p <* .001; Figure 8c) transects. Intra‐seasonal shifts for lower wing spot totals are therefore predicted across the country, regardless of location.

**FIGURE 9:**
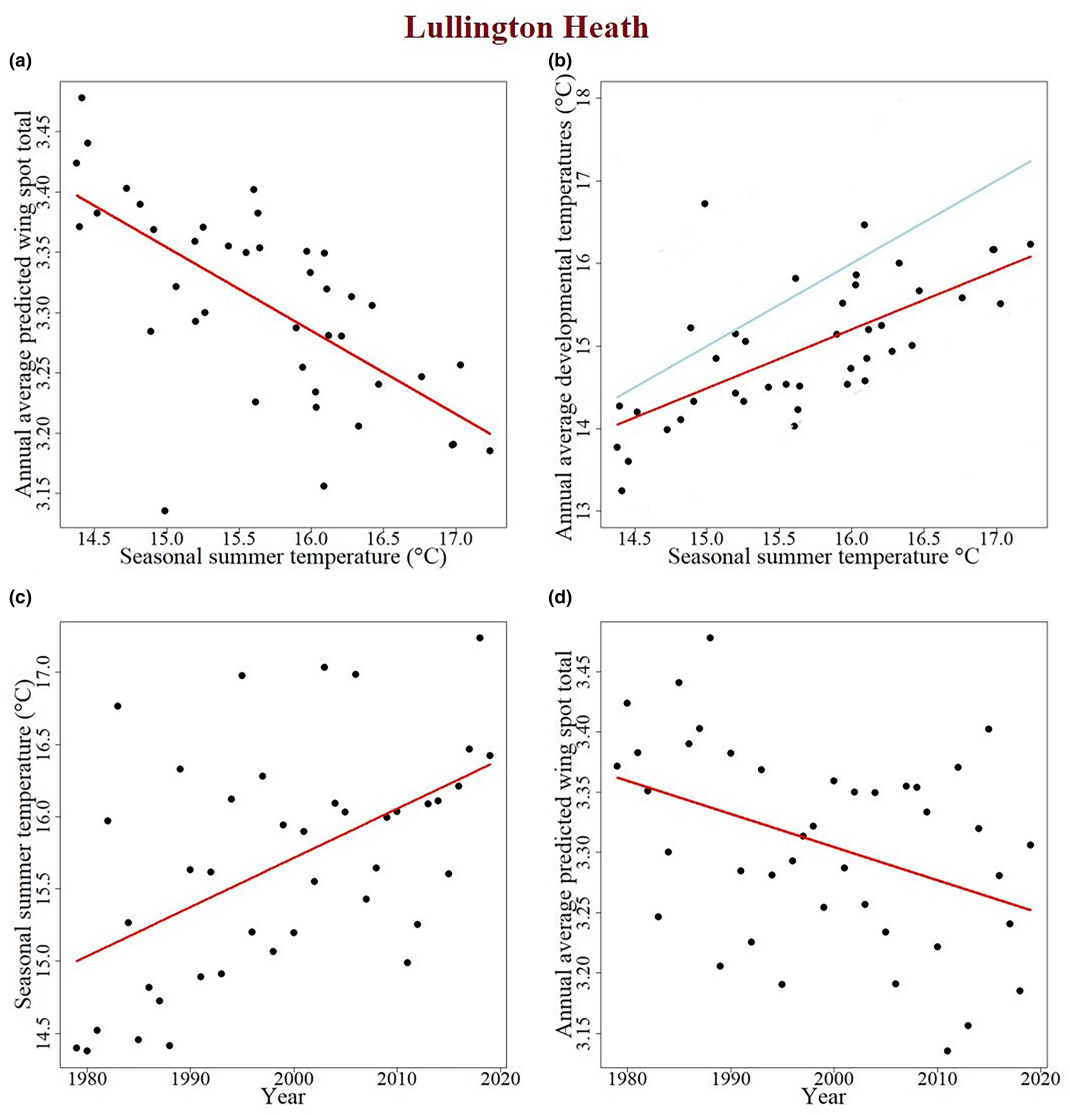
Temperature data over multiple years for a single site in Southern England, Lullington Heath, predicts a long‐term decline in female spottiness. (a) Average predicted wing spot total declines as seasonal summer temperatures increase. Each point shows a different year the transect was walked, and the red regression line shows the model. (b) Annual average developmental temperature increases as seasonal summer temperatures increase. The red line shows the slope of the linear model for the relationship between the developmental and summer temperatures. Note, the slope of this line differs to that of a one‐to‐one ratio shown by the blue line (see text). (c) Seasonal summer temperatures have increased significantly over the 40 years the Lullington Heath transect has been walked. (d) The average predicted wing spot total declines over time with warmer summers.

To predict how increasing summer temperatures might decrease female spottiness we also looked at a single BMS transect from Lullington Heath, in the South of England. The seasonal summer temperature at this site is predicted to have a significant effect on annual average predicted wing spot total (GLM: $\chi_{3,1}^{2}$ = 24.5, *p <* .001; Figure 9a) with female spottiness decreasing with increasing temperature. The seasonal summer temperature is also correlated with annual average developmental temperature with developmental temperatures increasing as summer temperatures increase (GLM: $\chi_{3,1}^{2}$ = 25.2, *p <* .001; Figure 9b). However, there is considerable scatter within this relationship (Figure 9b) and seasonal temperature alone is therefore not expected to be a good predictor of developmental temperatures or spot pattern. Seasonal summer temperatures have increased significantly over the 40‐year span that the transect has been surveyed (GLM: $\chi_{3,1}^{2}$ = 12.8, *p <* .001; Figure 9c). Finally, therefore, year also has a significant effect on the annual average predicted wing spot total (GLM: $\chi_{3,1}^{2}$ = 7.40, *p =* .006; Figure 9d) with the annual average predicted wing spot total predicted to decrease over time. Taken together, these data show that seasonal summer temperature (calendar‐based recording of temperature) alone is a poor predictor of spot variation, suggesting that care needs to be taken when selecting the correct variable to predict the effects of climate warming on phenotypic change.

## 4 DISCUSSION

### 4.1 The developmental model and total spot count

Taken together, our results show that variation in the number of spots in Meadow Brown butterflies is correlated with field temperature during larval/pupal development, both within and between seasons. Previous authors have suggested that this classic polymorphism is generated by the differential survival or development of high and low spot ‘morphs’ (Baxter et al., 2017; Beaufoy et al., 1970; Bengston, 1978; Brakefield & van Noordwijk, 1985; Conradt et al., 2000; Creed et al., 1964; Dowdeswell et al., 1949, 1957, 1960; Dowdeswell & Ford, 1955; Dowdeswell & McWhirter, 1967; Frazer & Willcox, 1975; Grill et al., 2007; McWhirter, 1969; Scali, 1972). Limited laboratory experiments varying temperature during pupation have previously failed to show a role of temperature in spotting (Brakefield & van Noordwijk, 1985). However, the role of field temperature inferred here may help explain why drought or changes in grazing (with shorter grass increasing the temperature close to the ground) might affect total spotting. It may also provide an alternative hypothesis as to why spot number appears to vary on either side of single hedgerow (Creed et al., 1959), as different aspects of a hedgerow will differ in their microclimate.

Our finding that the time window that best correlates with spot number is 35 days prior to capture (Figure 1c) is consistent with the butterfly spending 28 days in the pupa (Dowdeswell, 1981; Eeles, 2019) and ~7 days on the wing prior to capture, placing this time window at the anticipated point of late larval development or early pupation, which is the critical developmental period for butterfly wing pattern determination (Beldade et al., 2002; Beldade & Brakefield, 2002). This observation is also consistent with a recent comparative study of eyespot variation in other satyrid butterflies, where temperature has also been shown to drive eyespot size variation via the action of the insect hormone 20‐hydroxyecdysone (Bhardwaj et al., 2020). Clearly as our results are still correlative, so we cannot accurately say that our developmental time window corresponds to any specific larval or pupal stage of the butterfly which would require the study of caged larvae in the field. We also cannot guarantee the precise age (time since emergence) of any of the adults we have collected. Finally, we have used remote recordings of temperature and have not recorded temperatures in the field sites themselves. However, the intensive daily nature of collection at all sites, in all years, ensures that the adults we have collected are as close to emergence as possible and therefore are all a similar age as possible in a real field situation. The unique lifestyle of the Meadow Brown, whereby larvae, pupae and adults (all constantly developing, pupating and hatching at the same time) can all be found over a single generation for 2 to 3 months therefore makes such an analysis feasible.

### 4.2 Developmental temperature and the presence/absence of individual spots

When we look at the effect of temperature on each individual female spot‐position intriguingly we find that only those spots that are visible to predators, when the butterfly is at rest, vary in their presence/absence with developmental temperature and the spots that are ‘hidden’ (not visible from the underside due to the overlap of hindwing with the forewing) do not vary significantly (compare Figure 6b with c). These data are consistent with the hypothesis that spots visible in the female at rest are under temperature‐related developmental control and that an increase in temperature across the summer drives the decline in the spottiness of females throughout the season. These data are also consistent with the hypothesis, derived from the tropical butterfly *Bicyclus anynana* (Lyytinen et al., 2004), that predation maintains eyespot plasticity. It is also consistent with the idea that ‘covered’ eyespots, which are not continuously visible to predators, are less likely to be under positive selection (Chan et al., 2021). Furthermore, the observation that spottiness in males, unlike females, is not under such strong temperature‐driven control probably explains why E.B. Ford and others only examined variation in females (Dowdeswell, 1981; Dowdeswell et al., 1949, 1957, 1960; Dowdeswell & Ford, 1955; Dowdeswell & McWhirter, 1967), as these are indeed more variable (plastic) in the field. Most of the variation in male spots in our study is therefore associated with between site variation. In contrast to the presence–absence of the other smaller spots, the size of the large compound spot 2/3 is best explained by day of the year (days since 1st March) and the size of this large spot scales with wing length, as do eyespots in some other species (Brakefield et al., 1996). However, fascinatingly, this omni‐present spot appears to be under different developmental control (possibly photoperiod), perhaps because it is always required to startle predators while the butterfly is stationary and therefore it is always present at all developmental temperatures. Taken together, this suggests that reduced visible spotting in resting females may improve crypsis (Dennis & Shreeve, 1989) whereas the apparent insensitivity of male spotting to temperature might suggest they are more important in male–female signalling and thus sexual selection.

### 4.3 The phenology of spot variation across the United Kingdom

Finally, in order to predict how spot number will decline in the face of warmer summers we modelled the effects of increasing summer temperatures both at a single site, Lullington Heath (Figure 9), and across a series of UK BMS transects combined (Figure 8). Strikingly, our developmental temperature model predicts that our warmer summers will indeed lead to less spotty females (both within and between years) but, surprisingly they also show that average temperatures across the season are a poor predictor of developmental temperatures (within the key window of spot determination) and are therefore not likely to predict spot variation when viewed on their own. This demonstration of temperature‐related phenotypic plasticity stands in contrast to previous work predicting that spot variation is explained by population genetics (differential selection on high‐ and low‐spot morphs). Thus, if spotting declines as a function of increased temperature then it is not necessary to postulate complex explanations of the differential fitness of high‐ and low‐spot morphs in order to explain spot variation. Furthermore, it also suggests that our warming climate will drive further loss of butterfly spottiness year on year across the United Kingdom.

## AUTHOR CONTRIBUTIONS

**Sophie Mowbray:** Conceptualization (equal); data curation (equal); formal analysis (equal); writing – original draft (equal); writing – review and editing (equal). **Jonathan Bennie:** Software (equal); supervision (equal); validation (equal); visualization (equal); writing – original draft (equal). **Marcus W. Rhodes:** Data curation (equal); formal analysis (equal). **David A. S. Smith:** Conceptualization (equal); data curation (equal); formal analysis (equal). **Richard H. ffrench‐Constant:** Conceptualization (equal); data curation (equal); formal analysis (equal); funding acquisition (equal); writing – original draft (equal); writing – review and editing (equal).

## FUNDING INFORMATION

This work was supported by the Biotechnology and Biological Sciences Research Council (grant number BB/H014357/1) and a Royal Society Merit Award to R.ff‐C held at the University of Exeter.

## CONFLICT OF INTEREST STATEMENT

The authors declare no competing interests.

## Data Availability

Data and R codes are available at FigShare 10.6084/m9.figshare.22759490.
